# Case Report: Cervical approach for dual mediastinal ectopic thyroid: clinical utility of 3D imaging in surgical planning

**DOI:** 10.3389/fsurg.2026.1789284

**Published:** 2026-05-11

**Authors:** Chun-Miao Tsai, Shih-Ming Huang, Chia-Ying Li, Li-Hsun Chen

**Affiliations:** 1 Asian International Thyroid Center (AITC), Chang Bing Show Chwan Memorial Hospital, Changhua County, Taiwan; 2 Department of Chest Surgery, Chang Bing Show Chwan Memorial Hospital, Changhua County, Taiwan; 3 Asian Institute of Telesurgery (IRCAD-Taiwan), Chang Bing Show Chwan Memorial Hospital, Changhua County, Taiwan

**Keywords:** 3D reconstruction, airway compression, cervical approach, mediastinal ectopic thyroid tissue, primary hyperparathyroidism, VR-RENDER

## Abstract

We report an exceptionally rare case of synchronous dual ectopic thyroid tissue (ETT) located in both the anterior and posterior mediastinum of a 54-year-old woman presenting with a symptomatic multinodular goiter and early-stage primary hyperparathyroidism. Preoperative evaluation revealed significant tracheal compression and deviation. The patient underwent a combined procedure consisting of right subtotal thyroidectomy, left total thyroidectomy, and parathyroidectomy. While thoracoscopic resection was initially considered for the mediastinal masses, three-dimensional (3D) vascular reconstruction (VR-RENDER) revealed that the blood supply originated exclusively from cervical vessels, with no identifiable thoracic pedicles. This crucial finding enabled the safe extraction of both isolated mediastinal lesions via a single transcervical incision using blunt manual dissection, thereby obviating the need for sternotomy or thoracoscopy. The surgical outcome was excellent, with minimal blood loss (< 5 mL) and no postoperative complications. This case highlights the complex diagnostic and surgical challenges of multi-compartmental ETT and underscores the critical role of 3D imaging in optimizing preoperative planning and facilitating minimally invasive surgical approaches.

## 1 Introduction

Ectopic thyroid tissue (ETT) is a developmental anomaly characterized by the presence of thyroid tissue outside its normal pre-tracheal location, with the lingual region being the most common site (1, 2). Mediastinal ETT is exceedingly rare, representing less than 1% of all ectopic cases, and its occurrence in the posterior mediastinum is even more exceptional (3). Although many patients remain asymptomatic, mediastinal ETT can cause significant compressive symptoms, such as dyspnea or dysphagia, or may be discovered incidentally during imaging for unrelated conditions.

The management of mediastinal ETT presents a significant surgical challenge, particularly in determining the optimal operative approach—transcervical, sternotomy, or thoracoscopy (VATS). Traditionally, the choice of approach depends on the tumor's location and its relationship to major intrathoracic structures. However, preoperative identification of the primary vascular supply is often the decisive factor in ensuring a safe resection and minimizing blood loss.

We present a rare case of synchronous, anatomically distinct ETT located in both the anterior and posterior mediastinum in a patient with a symptomatic multinodular goiter and early-stage primary hyperparathyroidism (PHPT). Preoperative three-dimensional (3D) reconstruction and vascular mapping were instrumental in delineating the lesions’ complex anatomical relationships and confirming a cervical origin of blood flow. This advanced imaging strategy guided the surgical team toward a single, minimally invasive transcervical approach, enabling the complete resection of both mediastinal tumors and the hyperfunctional parathyroid gland without the morbidity associated with sternotomy or thoracoscopy.

## 2 Case presentation

A 54-year-old woman with a five-year history of a gradually enlarging right-sided neck mass was referred for evaluation after a routine health checkup revealed significant tracheal compression. She denied any history of radiation exposure or familial endocrine disorders. Her primary symptom was mild dysphagia; however, she reported no exertional dyspnea or voice changes. The patient expressed a strong preference for a minimally invasive approach to resolve the compression while minimizing surgical trauma.

A timeline table including diagnostic imaging, laboratory testing, scintigraphy, surgery date, pathology results, and postoperative follow-up milestones is shown in Table 1. The patient was hospitalized on March 20, 2025, and underwent surgery on March 21, 2025. Preoperative evaluation utilized contrast-enhanced CT (0.625 mm slice thickness), with DICOM data processed via VR-RENDER Fusion software (version 1.0, IRCAD, Strasbourg, France). Preoperative 3DCT imaging indicated that the mediastinal lesions were situated within 10 cm below the thoracic inlet. This volume-rendering platform, previously validated by our team for complex endocrine resections, enabled the creation of a high-fidelity 3D anatomical model. The software integrates these slices to create a 3D volumetric model, allowing for 360-degree rotation and layer-by-layer vascular dissection. Prepared by senior radiographers at IRCAD-Taiwan (processing time: 120–240 min), this ’spatial roadmap’ delineated the critical relationship between the ectopic masses and the cervicothoracic vasculature (4).

**Table 1:** Timeline including: detection, diagnostic imaging, laboratory testing, scintigraphy, MDT interpretation, decision-making, surgery date, pathology results, and postoperative follow-up milestones.

Category	Time/Clinical Detail
Hospitalization	March 20, 2025
Diagnostic imaging	contrast-enhanced CT (0.625 mm slice thickness) with DICOM data processed via VR-RENDER Fusion software
Tc-99 m pertechnetate thyroid scintigraphy	cold nodules in the lower poles of both thyroid lobes
Preoperative laboratory testing	confirmed a euthyroid state (T3: 127.3 ng/dL, free T4: 1.33 ng/dL, TSH: 0.202 μIU/mL) but revealed elevated thyroglobulin (197.7 ng/mL), serum calcium (10.1 mg/dL), and intact parathyroid hormone (iPTH: 56.99 pg/mL)
Surgery	March 21, 2025
Pathology results	benign multinodular goiter in both cervical thyroid lobes and non-neoplastic, benign ETT in both the anterior and posterior mediastinal tumors. The resected parathyroid gland showed diffuse chief cell hyperplasia.
Postoperative biochemical course	a transient elevation in iPTH on postoperative day 1, followed by a stabilization to baseline reference levels by postoperative day 2. The patient's serum calcium normalized postoperatively.
Discharged from hospital	March 24, 2025
Follow-up duration	From March 2025 to present (approx. 9 months), every 3 months
Medication	Levothyroxine (Eltroxin) 100–250 mcg daily
Functional outcomes:	
Laboratory trends	TSH, serum calcium, and iPTH stabilized within normal limits
Imaging (Ultrasound)	No recurrence of mediastinal or cervical lesions
Symptom resolution	Complete resolution of dysphagia; no airway symptoms
Complications	Clavien-Dindo Grade 0 (No postoperative complications)

**Figure 1:**
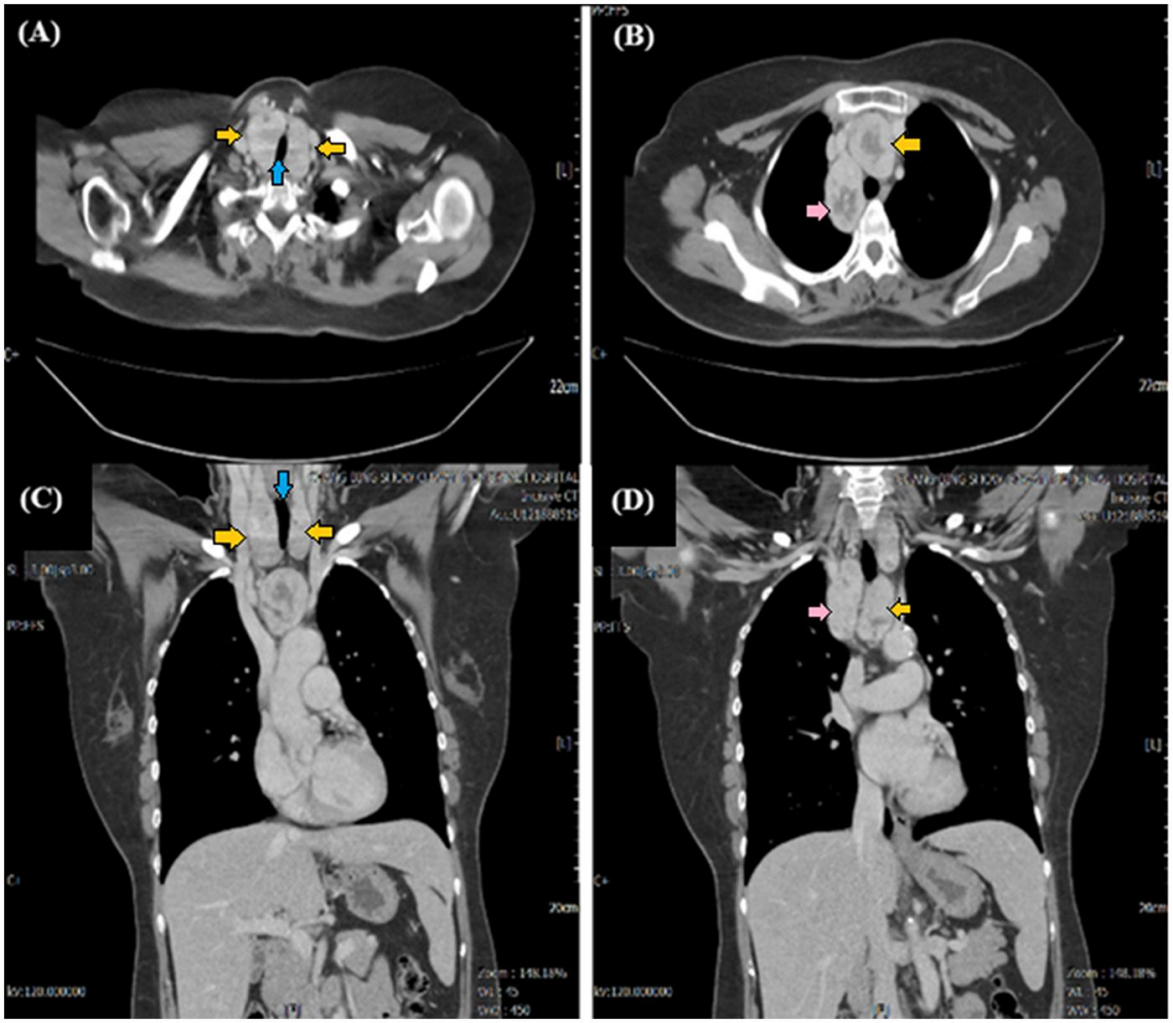
Preoperative contrast-enhanced CT and anatomical correlation. Axial **(A)** and coronal **(C)** CT views of the neck demonstrate a massive bilateral multinodular goiter (yellow arrows) causing significant displacement and high-grade compression of the trachea (blue arrow). Axial **(B)** and coronal **(D)** CT views of the chest reveal two well-circumscribed, anatomically isolated mediastinal masses distinct from the cervical thyroid gland. One lesion is situated in the pre-tracheal anterior mediastinum (yellow arrow), while the other is located in the retro-esophageal posterior mediastinum (pink arrow). Scale bars are provided for anatomical orientation.

**Figure 2:**
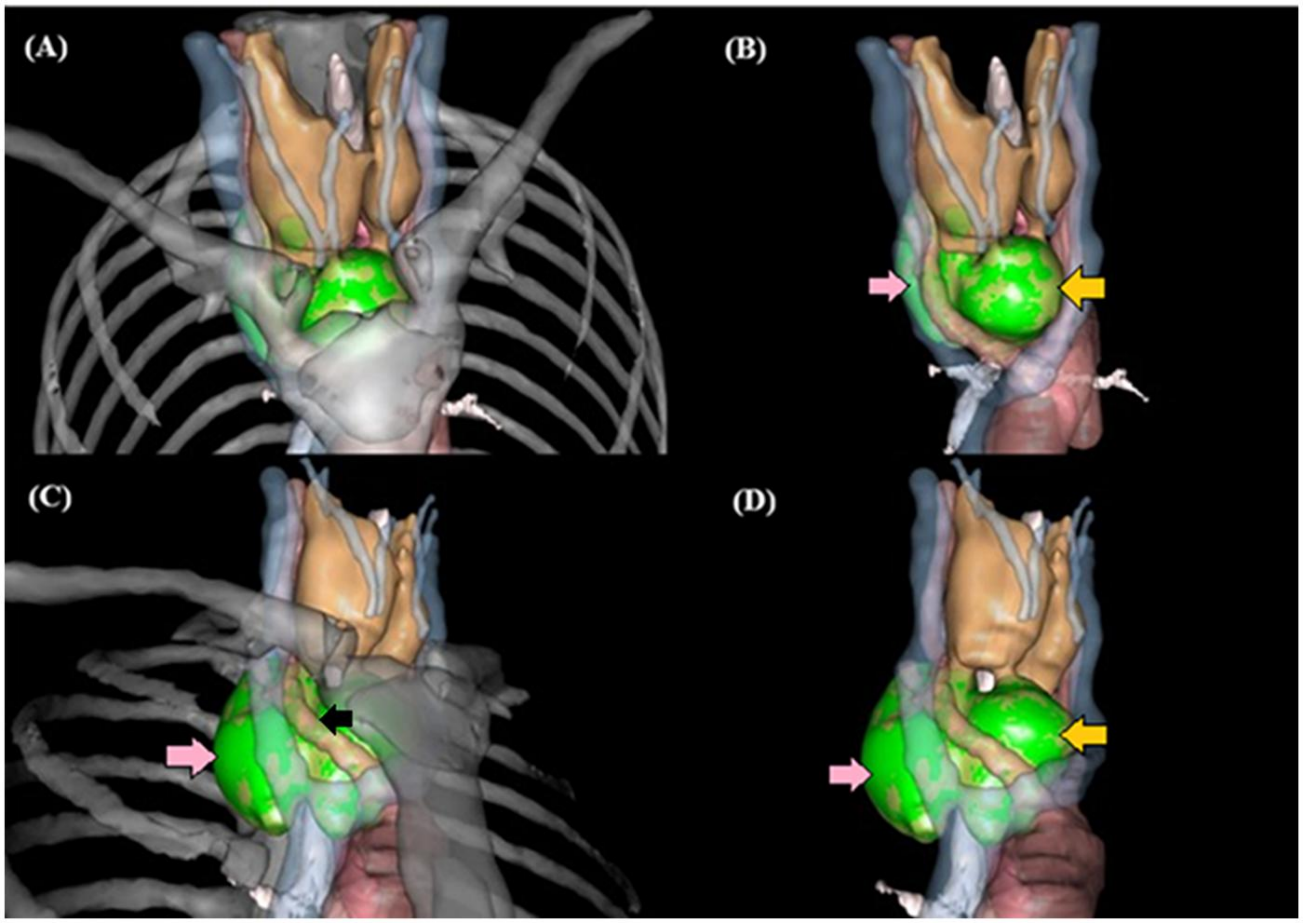
3D anatomical volume rendering and spatial mapping of dual-site ETT. **(A)** 3D spatial mapping provides a comprehensive overview of the dual ectopic lesions; green structures explicitly denote the ETT to distinguish them from the surrounding anatomy. **(B)** Anterior view highlighting the upper-anterior mediastinal ETT (yellow arrow) situated in the pre-tracheal space; the posterolateral lesion (pink arrow) is partially visible in the background. **(C,D)** Postero-lateral view provides optimal visualization of the posterior mediastinal ETT (pink arrow) occupying the retro-esophageal corridor (indicated by the black arrow representing the esophagus). The high-fidelity 3D model confirms a complete absence of a parenchymal bridge or direct anatomical connection between the cervical thyroid gland and the isolated mediastinal masses, supporting the diagnosis of true ETT.

**Figure 3:**
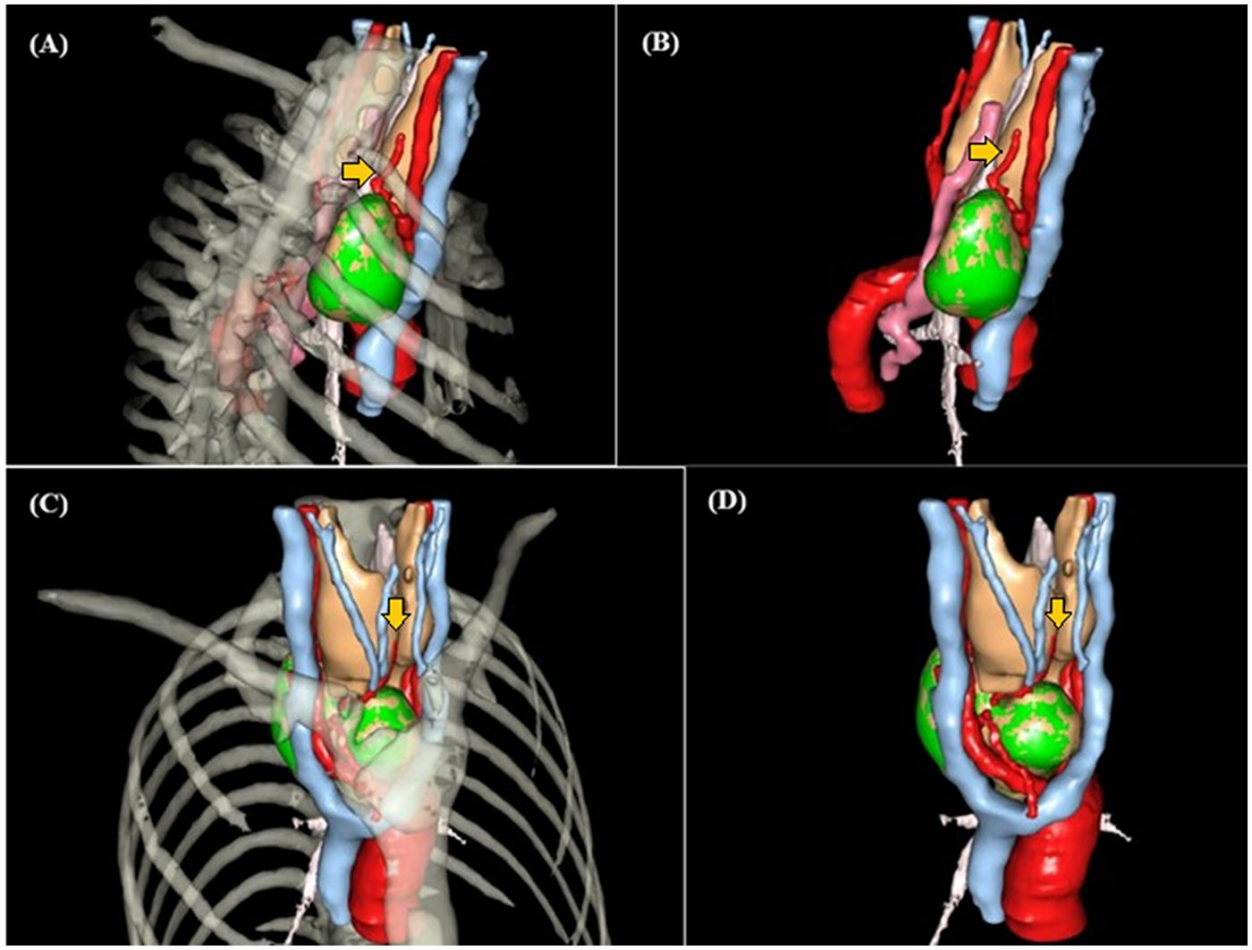
Vascular mapping and surgical planning via VR-RENDER. Detailed lateral **(A,B)** and anterior **(C,D)** views focused on vascular supply. Yellow arrows indicate the feeding vessels (inferior thyroid artery branches) descending from the cervical region to the mediastinal ETT. Green structures denote the isolated ectopic masses. No significant vascular supply from the thoracic aorta or internal thoracic arteries is observed.

The 3D reconstruction identified two anatomically isolated, well-defined mediastinal masses: a 6 × 6 × 7 cm lesion in the pre-tracheal anterior mediastinum and a 6 × 6 × 8 cm lesion in the retro-esophageal posterior mediastinum, both separated from the cervical thyroid by a distinct fat plane. The right nodular goiter measured 10 cm (approx. 120 g), and the left nodular goiter measured 8 cm (approx. 80 g) (Figures 1, 2). Crucially, 3D vascular mapping confirmed the absence of definitive arterial supply from the thoracic aorta or internal mammary arteries, indicating that perfusion descended from the cervical inferior thyroid arteries (Figure 3). This finding was pivotal for risk assessment, confirming that the tumors could be safely delivered via the thoracic inlet through a standard transverse neck incision, thereby avoiding more invasive sternotomy or thoracoscopy.

**Figure 4:**
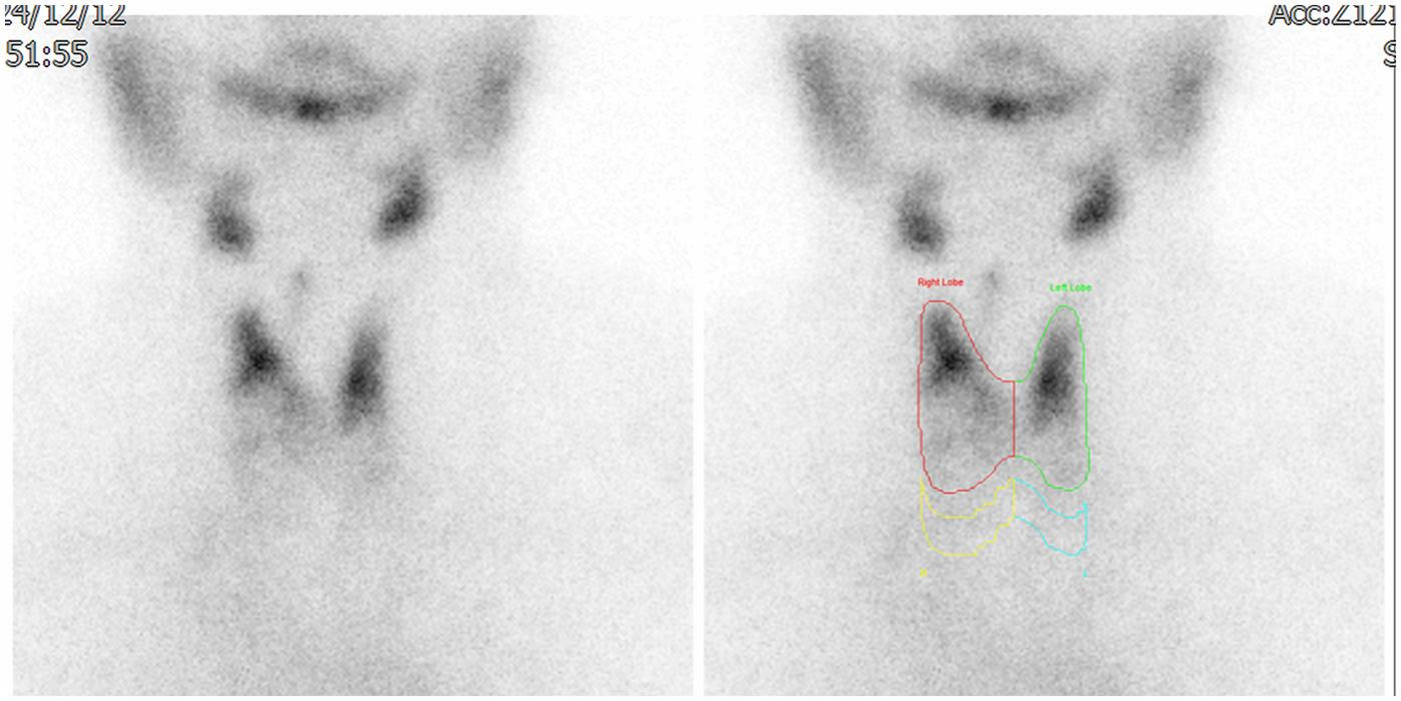
Tc-99 m pertechnetate thyroid scintigraphy. Anterior scintigraphic view demonstrates multiple “cold” nodules in the inferior poles of both thyroid lobes, with a markedly reduced total tracer uptake (0.6%; reference range: 0.5%–4.0%). No anomalous tracer accumulation is identified in the mediastinal regions, indicating that the ETT are non-functional. This lack of uptake, while confirming the non-autonomous nature of the masses, necessitated definitive histopathological confirmation via surgical resection due to the significant tracheal compression visualized on 3D imaging.

Tc-99 m pertechnetate thyroid scintigraphy showed cold nodules in the lower poles of both thyroid lobes, with no ectopic uptake in the mediastinum and a markedly reduced total thyroid uptake (0.6%) (Figure 4). Preoperative laboratory testing confirmed a euthyroid state (T3: 127.3 ng/dL, free T4: 1.33 ng/dL, TSH: 0.202 *μ*IU/mL) but revealed elevated thyroglobulin (197.7 ng/mL), serum calcium (10.1 mg/dL, Ref: 8.6–10.0 mg/dL), and intact parathyroid hormone (iPTH: 56.99 pg/mL). These findings, combined with normal vitamin D and serum creatinine levels, were consistent with early-stage normocalcemic PHPT.

The patient underwent right subtotal thyroidectomy (10 cm, 30 g nodule goiter), left total thyroidectomy (8 cm, 27 g nodule goiter), and right upper parathyroidectomy. The procedure was performed under general anesthesia with endotracheal intubation, with the patient in the supine position and the neck slightly extended. The mediastinal tumors were accessed via the thoracic inlet. Both mediastinal ETTs were successfully resected via the standard low collar transcervical incision using manual blunt dissection along the pre-vertebral and retro-esophageal planes (for the posterior/middle mediastinal tumor) and the pre-tracheal plane (for the anterior tumor), guided by continuous intraoperative neuromonitoring (C-IONM) to ensure the functional integrity of the recurrent laryngeal nerves (RLN) during deep dissection. Total operative time was 180 min. Total blood loss was minimal (< 5 mL), and no drains were required. A closed wound vacuum drain was placed before layered wound closure. No conversion to sternotomy or thoracotomy was required, validating the preoperative planning provided by the 3D imaging. The primary challenge was the limited visualization within the deep mediastinum; however, the use of C-IONM and the 3D-guided knowledge of the vascular anatomy mitigated the risk of nerve injury or uncontrollable hemorrhage. There were no intraoperative or postoperative complications. The patient exhibited no hoarseness or dysphagia immediately post-surgery. Following a smooth recovery (Clavien-Dindo Grade 0), a transient decrease in serum calcium (6.5 mg/dL) occurred on postoperative day 1 but was successfully managed with medication. Notably, the patient's intact PTH levels, which were at the borderline for hyperparathyroidism preoperatively, returned to the baseline reference range post-resection, confirming the resolution of the early-stage PHPT. The patient was discharged on postoperative day 3 (March 24, 2025). Since discharge, she has been followed up at 1, 3, and 6 months confirming stable laboratory values and no recurrence of symptoms. Periodic neck and upper chest ultrasound examinations have been performed during follow-up, confirming the absence of recurrence or residual ETT. At 9-month follow-up, her dysphagia had completely resolved, and laboratory values (TSH, calcium, and iPTH) remained stable under levothyroxine supplementation (100–250 mcg), indicating successful management of her thyroid and parathyroid status. The patient reported high satisfaction with the functional and cosmetic outcomes. The patient's preoperative symptoms, specifically the mild dysphagia, have completely resolved. She reports no new airway symptoms or voice changes, and her quality of life has significantly improved.

## 3 Surgical findings and pathology

Intraoperatively, both mediastinal masses were found to be encapsulated and anatomically distinct from the cervical thyroid. The right upper parathyroid gland was found to be hyperemic and significantly enlarged (60 mg), confirming the clinical suspicion of early-stage PHPT**.**

The final histopathological examination (H&E staining) demonstrated classic and definitive features of multinodular goiter and ectopic goiter, characterized by macrofollicles lined with flattened epithelium and filled with abundant colloid. Histopathologic examination confirmed benign multinodular goiter in both cervical thyroid lobes and non-neoplastic, benign ETT in both the anterior and posterior mediastinal tumors. The resected parathyroid gland showed diffuse chief cell hyperplasia, consistent with the preoperative diagnosis of PHPT. Importantly, microscopic evaluation confirmed R0 resection status (negative margins) for all specimens, with no evidence of atypia or malignancy observed. Given the highly characteristic morphological appearance and the lack of cellular atypia or suspicious architectural patterns, the attending pathologist determined that ancillary immunohistochemical (IHC) markers were unnecessary for confirming the diagnosis or ruling out malignancy.

## 4 Discussion

Dual ETT simultaneously occurring in the anterior and posterior mediastinum is an exceedingly rare clinical entity. To our knowledge, fewer than 10 such cases involving synchronous dual-site isolated mediastinal ETT have been documented in the English literature to date (3, 5). The novelty lies in the coexistence of three rare factors: 1. Synchronous dual-site involvement: Most reports describe either anterior OR posterior lesions; dual-site involvement is exceptionally rare. 2. “Isolated” but cervical-dependent: Although the masses were anatomically isolated (no tissue bridge to the neck), our 3D imaging confirmed they retained a cervical vascular supply, which is a rare physiological finding for deep mediastinal ETT. 3. Surgical minimalism: We successfully managed a posterior mediastinal mass—which typically necessitates a thoracic approach—through a single cervical incision (Table 2). The synchronous appearance in both mediastinal compartments suggests a complex embryological origin, potentially involving either the early fragmentation of the thyroid anlage during descent or the heterotopic differentiation of uncommitted endodermal cells in the primitive pharynx. While the anterior mediastinal mass likely reflects an over-descent or sequestration of the main thyroid anlage (primordium), the posterior (retro-esophageal) mass might be better explained by the differentiation of pluripotent endodermal cells located in the posterior aspect of the primitive pharynx. The spatial isolation of these masses, as confirmed by our 3D imaging (Figure 2), strongly hints that they were not merely extensions of the same migrating tissue, but rather independent embryological events. While often asymptomatic, ETT can present with compressive symptoms such as dysphagia or tracheal deviation (5–7). The differential diagnosis for mediastinal masses includes thymoma, lymphoma, germ cell tumors, and metastases (8). ETT should be considered when a well-defined, non-invasive lesion is located adjacent to the trachea, particularly when 3D imaging confirms a vascular connection to the cervical region (6, 9).

**Table 2:** Comparison of the present case with typical mediastinal ETT literature.

Feature	Conventional Mediastinal ETT	Our Case (Dual Mediastinal ETT)
Number of Lesions	Usually single lesion	Dual synchronous lesions (Anterior + Posterior)
Anatomic Location	Predominantly Anterior Mediastinum	Simultaneous Anterior and Posterior involvement
Vascular Origin	Often branches from Internal Mammary or Aorta	Exclusively Cervical-derived (Cervical Thyroid Vessels)
Surgical Approach	Sternotomy or Thoracotomy (for Posterior/Deep ETT)	Single Transcervical Incision (Cervical-only)
Clinical Value of Imaging	Diagnostic only (2D CT)	3D-guided Vascular Mapping for Surgical Planning

The clinical significance of this case is further highlighted by the atypical anatomical distribution and vascular patterns of the lesions, as summarized in Table 2. While the vast majority of mediastinal ETT cases involve a single lesion—predominantly in the anterior mediastinum (∼85%)—our patient presented with synchronous, anatomically isolated masses in both the anterior and posterior compartments. Notably, although preoperative Tc-99 m scintigraphy demonstrated non-functional (cold) mediastinal masses, the high-fidelity 3D reconstruction was the decisive factor in our diagnostic workup, as it accurately mapped the critical airway compression and the cervical-derived vascular supply.

A critical diagnostic challenge in mediastinal ETT is the identification of its vascular supply. Recent systematic reviews from 2023 to 2025 emphasize that the origin of vascular supply—cervical vs. thoracic—is the primary determinant in selecting the surgical approach (6, 10). In our case, advanced 3D vascular mapping revealed a purely cervical-derived supply from the inferior thyroid arteries, allowing us to safely bypass more invasive procedures (sternotomy or VATS) in favor of a single minimally invasive transcervical approach, effectively “delivering” the deep-seated posterior mass upward through the thoracic inlet. Unlike typical mediastinal tumors that receive flow from the internal mammary artery or aortic branches, our imaging revealed a downward-reaching vascular pedicle originating from the inferior thyroid artery (ITA). No definitive arterial branches were visualized arising from the thoracic aorta or its major intrathoracic branches.

The implementation of 3D reconstruction has recently been recognized as a “spatial roadmap” in endocrine surgery, significantly improving the localization of ectopic lesions and reducing the risk of injury to vital structures like the RLN (4). Preoperative 3D assessment identified two primary risks: (1) potential injury to the RLN due to the deep posterior location of the middle mediastinal mass, and (2) the risk of uncontrollable intrathoracic hemorrhage if a thoracic feeder existed. The imaging lowered the risk assessment by confirming the cervical vascular origin, which decreased the likelihood of needing a sternotomy. The 3D imaging directly guided the surgical plane by suggesting that the tumors could be “delivered” upward. Based on the vascular mapping, the surgical team prioritized a retro-thyroid and pre-vertebral dissection plane for the posterior mass, confident that the blood supply could be controlled from the neck. This guided the decision to use a single transcervical incision and blunt manual dissection rather than a more invasive thoracic approach. Vessels below the resolution of 0.625 mm CT (micro-vasculature) may not be visualized. However, for the purpose of identifying major surgical “danger zones”, the 3D VR-RENDER provided sufficient guidance. Furthermore, the incidental finding of early-stage PHPT in this patient adds a layer of clinical complexity. As noted by Cuhaci et al. (11), while 20%–50% of PHPT patients have coexisting thyroid disease, the association between PHPT and dual-site ETT remains under-investigated, necessitating a comprehensive preoperative biochemical workup.

While the scintigraphy demonstrated cold nodules and markedly reduced uptake (0.6%) without ectopic mediastinal uptake, these findings did not alter the primary surgical indication, which was driven by symptomatic tracheal compression and suspicious 3D imaging findings. However, the scintigraphy was essential for the preoperative differential diagnosis, helping to exclude functional autonomous thyroid tissue and further supporting the need for histopathological confirmation via surgical resection.

Regarding pathological confirmation, although specific IHC staining was not performed due to the definitive morphological features of benign multinodular goiter, we acknowledge the importance of markers such as thyroglobulin (TG), PAX8, and NKX2.1 (TTF-1) for excluding mimickers like thymic or germ cell tumors in ambiguous or suspicious cases (10, 12). Given that malignancy rates in ETT can reach 18.8%, achieving an R0 resection status remains the gold standard (10, 12). Our 9-month follow-up, characterized by stable laboratory values and a Clavien-Dindo Grade 0 outcome, validates the safety and oncological efficacy of this individualized, 3D-guided surgical strategy.

## 5 Conclusion

This case underscores the critical clinical significance of identifying synchronous dual-site ETT in atypical mediastinal locations. The integration of advanced 3D volume-rendering (VR) technology was instrumental in delineating the precise spatial relationship between the anatomically isolated anterior and posterior masses. By confirming a purely cervical-derived blood supply, this preoperative ’spatial roadmap’ enabled a safe, minimally invasive transcervical resection, successfully avoiding the morbidity associated with sternotomy or thoracoscopy. This case advocates for the wider adoption of patient-specific 3D reconstruction in managing complex mediastinal endocrine lesions.

## Data Availability

The original contributions presented in the study are included in the article/Supplementary Material, further inquiries can be directed to the corresponding author.
